# Perspectives of the Breast Cancer Survivorship Continuum: Diagnosis through 30 Months Post-Treatment

**DOI:** 10.3390/jpm5020174

**Published:** 2015-05-28

**Authors:** Jennifer M. Hulett, Jane M. Armer, Bob R. Stewart, Ausanee Wanchai

**Affiliations:** 1 Sinclair School of Nursing, Ellis Fischel Cancer Center, University of Missouri-Columbia, DC 116.05 Suite 408 Mizzou North, University of Missouri, Columbia, MO 65211, USA; E-Mails: armer@missouri.edu (J.M.A.); stewartb@missouri.edu (B.R.S.); wausanee@hotmail.com (A.W.); 2 Boromarajonani College of Nursing Buddhachinaraj, Muang, Phitsanulok 65000, Thailand

**Keywords:** breast cancer, health literacy, long-term survivorship, psychoneuroimmunology, self-empowerment, social support, spiritual/religious beliefs, worldview

## Abstract

This study explored breast cancer survivors’ perspectives regarding their experiences of the survivorship continuum from diagnosis through 30 months post-treatment. The sample included women (*N =* 379) with newly-diagnosed breast cancer undergoing treatment at a Midwestern university-affiliated cancer center. Semi-structured interviews were conducted using the Lymphedema and Breast Cancer Questionnaire at time of diagnosis, post-operatively, quarterly during the first year, and then semi-annually thereafter through 30 months post-treatment. A mixed-methodology was used to analyze participants’ comments. Themes central to long-term survivorship experiences included social support, positive worldviews, breast cancer and lymphedema health literacy, religious/spiritual beliefs, self-empowerment, and recovery expectations. These themes were consistent with a psychoneuroimmunological model of health in which psychosocial variables mediate stress and influence health outcomes. Qualitative data showed that social support and positive worldviews were the two themes with the most significant impact on long-term breast cancer survivorship experiences. Survivors expressed a need to advance their health care literacy in order to share ownership of breast cancer and lymphedema treatment decisions. Since breast cancer is an immune-mediated disease, long-term survivorship planning should address psychosocial factors that influence the long-term psychological distress associated with immune dysfunction.

## 1. Introduction

Breast cancer affects one in eight American women and is the second most common form of cancer among women worldwide. In the United States (U.S.), the population of long-term breast cancer survivors continues to grow with an estimated 2.9 million survivors. The most recently reported (1998–2003) relative five-year survival rates are the highest ever reported at 89.9 percent. The relative five-year survival rate is defined as a cancer survivors’ chance of survival (excluding all other causes of death than cancer) over a five-year period, compared to that of an average person (same age and same sex) [1,2,3]. Increased breast cancer survivorship rates have been attributed to improvement in detection methods, treatments, and supportive care measures [3]. However, a limited understanding exists regarding the experiences long-term survivors face, as longitudinal studies exploring this phenomenon have been few. The purpose of this paper is to disseminate findings from our 30-month study in which breast cancer survivors were interviewed regarding their perspectives of breast cancer survivorship and lymphedema.

In a systematic review, previous data showed that long-term physical symptoms among breast, gynecological, prostate, and rectal/colon cancer survivors included: pain, fatigue, sleep disorders, cognitive limitations, and sexual dysfunction [4]. In addition to physical function decline, breast cancer survivors experience poorer mental health and social difficulties in the post-treatment period [5,6,7,8]. A longitudinal study of breast cancer survivors (*N =* 312) followed over ten years post-diagnosis reported survivors experience poorer long-term general and physical health, more chronic illness, and decreased psychosocial well-being [9]. These findings were consistent with those of Schmidt [10] who reported a poorer quality of life among survivors with long-term fatigue (*N =* 1,928) at six years post-diagnosis with breast cancer.

Psychological distress has been reported by breast cancer survivors as early as the pre-biopsy stage, continuing throughout the post-treatment period [11]. Fear of breast cancer recurrence and the development of lymphedema have been the two most common sequelae of breast cancer treatment that persist throughout the survivorship trajectory [12,13,14]. Post-treatment lymphedema has an associated life-time risk and may affect 43% to 94% of breast cancer survivors within five years post-treatment for breast cancer, depending on the criteria applied and length of follow-up [15]. Lymphedema alone has been associated with long-term physical discomfort, which contributes to persistent psychological distress, negative self-image, depression, anxiety, diminished coping skills, and social isolation [7,16,17].

Rehabilitation from breast cancer treatment significantly differs from the experiences of other cancer populations because the recovery tends to be unpredictable and complicated by treatment-related late effects (e.g., lymphedema) [18]. Breast cancer survivors with lymphedema have reported increased frustrations with a medical community that conveys disinterest or lacks knowledge in addressing lymphedema signs and symptoms [19,20,21]. These experiences may help explain recent data that showed up to 91% of patients with advanced cancer engage in complementary alternative medicine (CAM) practices including prayer, relaxation, and exercise [22]. Among breast cancer survivors, CAM practices are influenced by pre-existing knowledge of CAM benefits to health, economic factors, social support benefits, and spiritual beliefs [23]. Data have also shown that chronically ill individuals rely on spiritual beliefs to reduce stress when experiencing life-threatening illnesses such as breast cancer [24,25]. In a systematic review, Schreiber and Brockopp [26] found that perceptions of well-being among female breast cancer survivors were associated with religious and spiritual beliefs. Among African-American breast cancer survivors and their caregivers, spiritual and religious practices and maintaining positive attitudes were viewed as essential for recovery from breast cancer [27].

The purpose of this paper is to analyze and disseminate qualitative data findings from the first 30 months of the Missouri Breast Cancer and Lymphedema Project, which was conducted from 2001 to 2010 [15,28]. Analysis of the data was conducted in order to gather a better understanding of breast cancer survivors’ perspectives regarding their long-term survivorship experiences of breast cancer and lymphedema. Data were derived from the longitudinal parent study which reported on the occurrence of breast cancer-related lymphedema in breast cancer survivors from diagnosis through 30 months post-treatment and have been reported elsewhere [28,29]. This study focused on a secondary aim of the parent study, which was to examine qualitative data collected from breast cancer survivor participants in order to uncover potentially new insights from survivors who were navigating an extended survivorship continuum.

## 2. Methods

### 2.1. Design and Setting

The parent study utilized a prospective, longitudinal design with repeated measures [28]. This study offers a primary analysis of the qualitative data from the parent study using a mixed-methodology. Initial approval for the data collection and analysis was obtained from the University of Missouri Health Sciences Institutional Review Board prior to commencement of the study. Recruitment, consent, enrollment, and participant interviews were conducted at Ellis Fischel Cancer Center at the University of Missouri-Columbia.

### 2.2. Participants

The sample (*N =* 379) included women older than 18 years of age, newly diagnosed with their initial breast cancer (stages I–IV), and no previous history of primary or secondary lymphedema. Participants previously received surgical treatments, including mastectomy and/or lumpectomy, sentinel lymph node biopsy, and/or axillary lymph node dissection, as well as post-surgical treatments including chemotherapy, radiation, and reconstructive surgery. Sample demographics are listed in Table 1 and have been reported with more detailed descriptions from the parent study elsewhere [28,29]. Survivors were up to 2.5 years post-diagnosis and treatment. The average stage of survivorship was not calculated due to serial responses by participants over the study timeline and differing exit points during the study due to death or relocation.

**Table 1:** Baseline Demographics ¹.

	(*N =* 379)	
Variable	Number	Percent
Median Age	57.9 years	
Age Range	20–92 years	
Survivorship Range	0–2.5 years	
Race/Ethnicity		
Caucasian	353	93.1
Other	10	2.6
Missing	16	4.2
Marital Status		
Married	248	65.4
Divorced/Separated	38	10.0
Single	24	6.3
Widowed	42	11.1
Missing	27	7.1
Education		
High School and below	41	10.8
Some college	213	56.2
College graduate	66	17.4
Graduate school	59	15.6

¹ Data extrapolated from parent study demographics reported by Cormier *et al.* [29].

### 2.3. Measures and Data Collection

Qualitative data were collected utilizing the Lymphedema and Breast Cancer Questionnaire (LBCQ). The LBCQ is a 58-item instrument that measures 19 symptoms associated with breast cancer treatment and post-treatment-related lymphedema. The LBCQ has a high measure of internal consistency (r = 0.785) and a high test-retest reliability (r = 0.98) [30]. The LBCQ provides for self-reporting of treatment history, disease course, demographic data, and post-treatment symptom experiences occurring currently or within the past year. The focus of this study was to analyze data elicited from one item on the LBCQ, question #58, which states, “If you have comments you would like to make about breast cancer and lymphedema, please use this space to share them with us.” Comments received from participants were strictly voluntary and did not utilize formal or structured answer options (e.g., multiple-choice).

Study enrollment began during the pre-operative period (*n =* 236) or within 30 days post-operative (*n =* 143) for breast cancer treatment. A trained research nurse-team administered the LBCQ to each participant in the laboratory setting during scheduled visits from time of diagnosis with breast cancer or immediately post-operative. Each participant was interviewed with the LBCQ at subsequent visits at 1, 3, 6, 9, 12, 18, 24, and 30 months post-treatment. LBCQ qualitative data were also collected by home completion and mail-back of the LBCQ at 1, 12, and 24 months postoperatively. Throughout the 30-month data collection period, each participant had at least eight opportunities (*i.e.*, 1-, 3-, 6-, 9-, 12-, 18-, 24-, and 30-months post-operative) to complete the LBCQ. Participants’ responses were transcribed and entered into an Excel spreadsheet using a double-entry method. All survivor participants’ comments were included for analysis. Responses from each participant (*N =* 379) across the 30-month data-collection period were examined by individual and summarized as a single narrative for each participant (*i.e.*, 379 sets of 8 responses from the same participants throughout the entire 30-month study period).

### 2.4. Data Analysis

A mixed-methodology was chosen for data analysis since multiple approaches provide complementary aspects of both quantitative and qualitative methods, as well as minimize weaknesses associated with using a single research method [31]. Strauss’ and Corbin’s [32] grounded theory method was selected for uncovering emerging patterns and themes from the data. Each individual participant’s responses for the 30-month study period was collected and examined during the initial coding process. First cycle coding was performed using *in vivo* coding in which data were sorted and assembled manually into a manageable list of words or phrases. During *in vivo* coding, simultaneous reflexive memo writing was also utilized to clarify and summarize participants’ responses into commonly used words, phrases, or concepts [33,34,35]. Participants’ comments were noted for the purpose of indexing and highlighting responses that might warrant additional analysis. Examples of *in vivo* codes include: “get a second opinion,” and “keep a positive attitude.”

During the first cycle *in vivo* coding, it was observed that participants often used wording and phrases to describe their survivorship experiences that were similar to those of other study participants. In order to be reflexive to this emerging trend, the research team elected to add a quantitative approach to the data analysis. An Excel spreadsheet was created to simultaneously track and match participants’ responses to the words or phrases already expressed by other participants. The frequency of responses was utilized to triangulate the qualitative interpretation of the codes and assist in second cycle coding which identified patterns of words and phrases [35].

Axial coding was selected as an additional coding method to extend the data analysis after pattern coding and achieve saturation [34]. This process facilitated the organization of major patterns into theme categories, subcategories, and relationships between the theme categories [33,34]. These categories were compared and further narrowed by similarities into six central themes. In keeping with the grounded theory tradition, the determination of themes was developed from the most commonly recurring patterns, while remaining mindful of the intensity and salience of participants’ comments. Additionally, a peer-review of pattern codes and themes was performed by two members of the investigative team who were in agreement that the final central themes adequately represented the data.

## 3. Results

Six themes were uncovered that reflected breast cancer participants’ perspectives regarding the factors that influenced their survivorship experiences: (1) social support; (2) worldview; (3) breast cancer and lymphedema health literacy; (4) religious and spiritual beliefs; (5) self-empowerment; and (6) recovery expectations. Table 2 presents the major themes, pattern codes, and frequencies of recurring pattern codes, which reflected the commonly reported survivor perspectives. Although the frequencies of responses were included, it should be emphasized that participants’ responses were spontaneous, voluntary, and obtained individually. Therefore, a low-frequency response is not necessarily less significant in the qualitative tradition, as the primary purpose of this study was to better understand long-term perspectives of breast cancer survivors, including the less-often mentioned comments that may be quite relevant.

**Table 2:** Patterns and Themes of Survivors’ Perspectives.

Theme	Pattern	Number of Participant Comments (n)	Percentage of Comments (%)
Social Support	Social support, including spouse, family, and friends is essential	192	50.7
	Talking about cancer experience helps relieve stress	44	11.6
	Organizational support including cancer center, cancer support group, and church group	77	22.1
	Pets provide comfort	6	1.6
Worldview	Keeping a positive attitude is essential to recovery	140	36.9
	Life goes on, go about your routine, stay active	69	18.2
	Don’t stress, evaluate your priorities	29	7.7
	Hang in there	17	4.5
	Used meditation, affirmations, imagery to cope	7	1.8
	Would have helped to be mentored by a survivor	5	1.3
Breast Cancer and Lymphedema Health Literacy	Regular mammograms and annual examinations are important	109	28.8
	Find breast lumps early, check any new symptoms, monthly breast exams important	104	27.4
	Don’t rely on a negative mammogram, get a second opinion	53	14
	Experienced emotional distress with manner in which biopsy results were conveyed	26	7
	Monitor for Lymphedema, see a Lymphedema specialist	20	5.3
	Experienced emotional distress with delayed receipt of biopsy results	12	3
	Hormone replacement therapy viewed as cause of cancer	8	2.1
Self-Empowerment	Educate yourself, be well-informed of your treatment options	70	18.5
	Important to educate family and others about breast cancer	18	4.7
	Lacked adequate pre-operative teaching about diagnosis	11	2.9
	Post-operative care of affected arm is important	14	3.7
	Wear compression sleeve when flying	10	2.6
	Does not recall any pre-operative lymphedema teaching	10	2.6
	Was not prepared for post-mastectomy body appearance	9	2.6
	Learn lymphedema massage	7	1.8
	Lymphedema education is important	4	1.1
	Fears getting lymphedema	3	<1
Religious and Spiritual Beliefs	Important to have faith in God/higher power	75	19.8
	Prayer is important, received prayer support from others	52	13.7
	Have faith in your doctor	19	5.1
	Sense of “God watching over me”	11	2.9
	Helping attitude towards others helps recovery	6	1.6
Recovery Expectations	Important to follow doctors’ recommendations, post-treatment follow-up is important	46	12
	Start range of motion exercises early after surgery	40	10.6
	Unhappy with lack of treatment options offered	26	6.9
	Make sure you have a good doctor	22	5.8
	Take it one day at a time	21	5.5
	Eat well, nutrition is important	18	4.7
	Learn to ask for help	18	4.7
	Breast cancer experience was not as bad as expected or not as bad as “others”	14	3.7
	Fatigue is problematic with trying to work	11	2.9
	Cancer “sucks”	10	2.6
	Breast cancer is emotionally draining	10	2.6
	Dissatisfied with physician communication	18	5.2
	Learn to accept help, pamper yourself	6	1.5

### 3.1. Social Support

A majority of participants (*n =* 192) (50.7%) commented on the importance of having social support from spouses, family, and friends. A common sentiment was, “I couldn’t have done it without the support of my family and friends.” Sources of social support often cited cancer centers, cancer support groups, church groups, and pets. These participants conveyed perceptions that their recovery experiences and emotional outlook would have been significantly worse, had they not received social support. Moreover, survivors (*n =* 44) (11.7%) shared that social support helped relieve survivors’ stress and that, “Talking about cancer helps relieve stress.”

### 3.2. Worldview

Survivors (*n =* 157) (41.4%) attributed keeping a positive attitude about life and a generally positive worldview as integral to surviving breast cancer. Examples of this sentiment included responses by participants that they held a belief that they would “beat cancer”. Advice offered by participants to other breast cancer patients included, “Have a positive attitude”, “Hang in there”, “Stay active”, and “Go on about your routine”. Respondents (*n =* 93) (24.5%) also advised that newly-diagnosed survivors should cope by focusing their attention beyond their breast cancer diagnosis and “Help others”, “Keep busy”, and “Get a hobby”, and understand that “Life goes on”. For patients newly diagnosed with breast cancer, survivors (*n =* 29) (7.7%) also advised, “Don’t stress” and “Evaluate your priorities”. A few survivors (*n =* 7) (1.8%) reported using complementary alternative medicine practices including relaxation, meditation, positive affirmations, visual imagery, and massage to maintain a positive outlook during survivorship.

### 3.3. Breast Cancer and Lymphedema Health Literacy

Breast cancer survivors (*n =* 109) (28.8%) reported that having regular mammograms and checkups was essential for early detection of breast cancer. Moreover, participants (*n =* 53) (14%) emphasized regularly monthly self-breast exams and early reporting of breast symptoms as another essential theme to successful breast cancer treatment. Participants also expressed wariness in trusting negative mammogram reports in the setting of a positive breast exam finding (e.g., lump) by stating, “Don’t trust a negative mammogram” and “Get a second opinion”. Comments regarding lymphedema detection and diagnostic practices were included by a small number of participants (*n =* 20) (5.3%) who stated, “Watch for lymphedema” and “Go to a lymphedema specialist”.

Additional comments regarding detection of breast cancer include a very small number of respondents (*n =* 8) (2.1%) who expressed regret in having used hormone replacement therapy and believed it to be the cause of their breast cancer. Very few survivors (*n =* 12) (3%) specifically mentioned experiencing emotional distress due to delayed notification of biopsy results. Similarly, a few survivors (*n =* 26) (7%) reported increased emotional distress due to their biopsy results being conveyed in an impersonal manner (e.g., voicemail), poor communication with providers, or perceptions of not receiving adequate information regarding treatment options.

### 3.4. Religious and Spiritual Beliefs

Participants (*n =* 94) (24.8%) expressed beliefs in God and having faith as being essential to their recovery. Among these comments, survivors (*n =* 75) (19.8%) attributed having beliefs of faith in God or a higher power as having intervened and facilitated healing. A common statement by survivors (*n =* 11) (2.9%) was, “God is watching over me”. Additionally, a few participants (*n =* 19, 5.1%) shared beliefs that having faith in their health care providers was important.

The theme of religious and spiritual beliefs also included perspectives from participants (*n =* 52, 13.7%) who reported relying on religious practices including prayer for healing. Moreover, survivors expressed feeling gratitude and being comforted by prayer practices and that prayers played a role in their recovery. Finally, subthemes related to spirituality were reported by survivors who shared perceptions of having “experienced cancer for a reason”, “having more patience”, “having compassion for others”, and “I think I am a better person since I had breast cancer”. Although a helping attitude is reported as a subtheme of positive attitudes and worldviews, some survivors discussed helping others from a compassionate and spiritual viewpoint stating, “Helping others helps recovery”.

### 3.5. Self-Empowerment

Participants (*n =* 70) (18.5%) commented that survivors needed to be well informed of treatment options and take initiative to educate themselves about breast cancer. Specifically, participants reported using online breast cancer website resources and breast cancer-related self-help books, discussions with friends, and seeking advice from other survivors as methods to feel more informed about their treatment decisions. Survivors also conveyed strong feelings regarding the importance of educating family members and the general public regarding the risks of breast cancer with comments, including “Educate yourself”, “Be well-informed”, and “Know your treatment options”. In some instances, survivors shared stories reflecting feeling remorse over their treatment options, stating they would have made a different choice had they realized how their treatment might impact future reconstructive breast surgery options.

### 3.6. Recovery Expectations

Survivors (*n =* 46) (12%) emphasized the importance of following physician treatment recommendations and completing post-treatment follow-up visits. Survivors noted that good nutritional habits and regular physical exercise were important during the recovery period; however, several participants noted that they did not receive any guidance on nutrition and exercise; and therefore, recommended self-help books that they had discovered on their own. Survivors also expressed beliefs that they benefited by starting range of motion exercises on the affected limb immediately in the post-operative period, as opposed to starting later in the recovery period as they had been advised by their health care providers.

There were a few comments from survivors (*n =* 18) (5.1%) regarding dissatisfaction with physician communication. These survivors reported perceptions of not having their concerns heard or addressed and stated, “Doctors need to listen”. Similarly, there was dissatisfaction with respect to receiving adequate information in order to make informed decisions regarding treatment options. This sentiment was accompanied with the advice, “Be sure you have a good doctor” (*n =* 22) (5.8%).

A small number of survivors with lymphedema emphasized the importance of educating all survivors about lymphedema risks and post-operative precautions with the limb at risk for lymphedema. A very small number of survivors (*n =* 11) (2.9%) expressed that they received inadequate teaching during the diagnosis and pre-operative period regarding breast-cancer treatment options, as well as recovery expectations. Similarly, only a few participants (*n =* 10) (2.6%) reported that they did not receive lymphedema teaching during the preoperative period. Moreover, these participants discussed feeling frustrated with their health care providers in failing to diagnose lymphedema, as well as adequately manage lymphedema symptoms. Only three survivors (<1%) expressed fears of developing lymphedema or experiencing progression of lymphedema.

Fatigue during recovery was a common problem, especially for participants (*n =* 11) (2.9%) who reported continuing to work post-treatment and throughout the recovery period. Additional perspectives related to the recovery period included survivors who reported that they chose to begin physical activity and range of motion exercises on their affected limbs before receiving approval from their health care providers. Moreover, a number of survivors also reported that they wished that they had started these exercises sooner. Survivors commonly offered advice to future survivors to understand that recovery takes time. Frequent suggestions related to allowing time for recovery included, “Learn to ask others for help” (*n =* 18) (4.7%), “Learn to accept help”, and “Pamper yourself” (*n =* 6) (1.6%). Another common suggestion from survivors (*n =* 21) (5.5%) was to “Take it one day at a time”.

The sentiments regarding survivors’ overall perceptions of their breast cancer post-treatment and recovery experiences were somewhat mixed. While very few respondents directly commented on their overall experiences of breast cancer; a few survivors (*n =* 14) (4%) expressed perceptions that their breast cancer experiences were not as bad compared to the experiences of other survivors with whom they were acquainted. Moreover, they expressed feeling grateful for many aspects of their treatment, recovery, and overall outcomes. A similar small group of respondents (*n =* 10) (2.8%) viewed their overall breast cancer experiences as emotionally draining and stated, “Cancer sucks”.

## 4. Discussion

The majority of participants shared that having positive social support systems was the most influential theme central to their survivorship. Social support generally refers to how an individual’s life is structured with respect to group memberships and familial relationships, as well as the purpose those structures provide to the individual (e.g., emotional benefits) [36]. Survivors consistently described receiving their social support from friends and family and that this support alleviated stress and psychological distress during recovery. Sixty-five percent of sample participants reported being married. Recent data suggested that being married was positively associated with cancer-related follow-up care use, decreased financial concerns, and better perceptions of overall health status [37]. Having a small social support network at five years post-treatment has been linked to poorer social functioning by 10 years post-treatment [9]. Moreover, the level of social support at time of diagnosis with breast cancer appeared to be predictive of post-treatment symptoms including pain, depression, and inflammation [38]. It is noteworthy that participation in the longitudinal parent study may have created a unique aspect of social support for this sample of survivors and, therefore, provided additional comfort to survivors as they progressed through the survivorship continuum.

The second most common theme expressed by participants was related to worldview. Worldview as a construct has multiple meanings, but in general, refers to a set of (background) assumptions that individuals use to give direction and purpose to life, as well as provide individuals with a set of values. Therefore, according to Stenmark [39], worldview may be defined as the “constellation of beliefs and values that (consciously or unconsciously) guide people in their attempt to deal with existential concerns” (p. 929). Survivors reported that keeping a positive attitude in the course of daily activities and maintaining a positive worldview helped them cope during their recovery from breast cancer. Survivors’ shared positive beliefs that they would be healed and held positive intentions to focus on the meaningful aspects of life, rather than focusing on having cancer. These attitudes suggested that a positive mindset or development of a deliberately positive worldview impacted their survivorship outcomes in a positive way.

Survivors reported positive spiritual and religious beliefs including subthemes of having selflessness and compassion for others. Spirituality has been described by many terms in the literature including: meaning, purpose in life, the mystical, the numinous, hope, value, optimism, emotional connectedness, transcendence, gratitude, and forgiveness [40,41,42]. In general, religion refers to a more external experience, defined and mediated by a group that follows a specific doctrine; while spirituality refers to a more personal experience with less doctrine association [43]. Survivors expressed “helping” attitudes, in which survivors coped with their diagnoses by shifting their focus to help others who appeared less fortunate to them. Expressions of gratitude toward God or a higher power, family and friends, and health care providers were evident in participants’ comments and reflective of spiritual and religious beliefs. Sentiments regarding an emotional connectedness to God or a higher power, helping others, and spiritual growth supports data from other survivors who also relied on spiritual and religious beliefs to make meaning of their breast cancer experiences [44]. Similarly, data from Whitford and Olver demonstrated that two terms often used to describe spiritual well-being, peace and meaning, were associated with quality of life perceptions among newly-diagnosed cancer patients [45]. Our data support findings by Patel [46] who reported that breast cancer survivors experience spiritual growth and development after experiencing cancer. Furthermore, the reliance on positive spiritual and religious beliefs relates to our worldview theme, suggesting these two variables were connected and inter-dependent.

A number of survivors recalled negative experiences navigating the health care system during the pre-diagnosis/biopsy stage. In the U.S., the health care professionals treating survivors during the pre-/intra-diagnosis stage would typically include: nurses, advanced practice nurses *(i.e.*, nurse practitioners and clinical nurse specialists), primary care physicians, surgeons, oncologists (*i.e.*, surgical, medical, and radiation), and physician assistants. Survivors discussed the impersonal manner in which they were notified of their biopsy findings (e.g., voicemail messages). Other survivors expressed increased psychological distress as a result of delayed biopsy results. While 30% of survivors felt screening mammograms were very important, a number of these participants also stated that they would distrust negative mammogram screening findings and recommended seeking a second opinion during the diagnostic process. These findings are consistent with a previous study conducted by Mellink [47], which showed that the majority of cancer patients seek second-opinions due to needs for reassurance and more certainty or because of dissatisfaction with their specialists.

While the overall health literacy of the cohort was not measured, demographic data show that the majority of participants were middle-aged, Caucasian, and have attended some college. This suggested a survivor sample with a higher educational level, potentially more diverse life experiences that come with age, and therefore, this sample may have a greater level of health care literacy. However, a number of survivor participants’ expressed an initial sense of feeling ill informed and powerless to make important treatment decisions regarding their breast cancer treatment during the early survivorship period. Those with lymphedema offered similar sentiments regarding inadequate teaching regarding arm care during the pre- and post-operative periods. These perceptions are important to consider in discharge planning because previous data have shown that cancer survivors with greater information needs have poorer perceptions of mental and physical health [48].

While survivors initially described feelings of powerlessness in early diagnosis and survivorship, comments over the 30-month data collection period suggested that some participants experience an increased sense of self-empowerment. Moreover, increasing self-empowerment suggests that a positive shift in worldview occurs during survivorship. Nearly 20% of survivor participants spontaneously commented that they took the initiative to do their own research regarding diagnostic testing and treatment options. These survivors conveyed a sense of responsibility for sorting through a variety of information sources beyond the information provided by health professionals (e.g., web-based resources) as a means to acquiring ownership of treatment decision-making. These findings provide insight regarding the importance of promoting breast cancer and lymphedema health literacy and the resulting implications this has for self-empowerment. Moreover, these findings are supported by previous data, which showed that cancer survivors have a desire to receive more information regarding testing, treatment, side effects, post-treatment symptoms, and health promotion practices [49]. Furthermore, feelings of self-empowerment may offer comfort to survivors by allowing them to perceive having a degree of control of an otherwise-unpredictable survivorship trajectory.

Perspectives regarding lymphedema monitoring and symptom management were infrequently reported in our study. In contrast with previous literature, very few survivors expressed fears of developing lymphedema or progression of lymphedema symptoms. It is possible that participation in the parent study may have influenced survivors’ perceptions and concerns regarding the development of lymphedema since the parent study included regular limb volume measures and lymphedema monitoring. Another implication is that survivors who did not have signs or symptoms of lymphedema remain unaware of this potential sequela of breast cancer treatment.

Themes related to the expectations of the recovery period centered on survivors learning to adjust daily habits and activities in order to allow time to heal. This included an acknowledgement that survivors need to plan for fatigue and embrace the concepts of asking and accepting help from others. Some survivors commented that guidance from primary health care providers was lacking regarding optimal nutrition and physical activity (*i.e.*, range of motion exercises for the same side, affected limb) in the post-treatment period. These sentiments reflected a common historical practice by U.S. health care providers in which the services of health psychologists, lymphedema therapists, and physical therapists were typically requested in the later stages of survivorship after late-treatment symptoms had developed. Survivors reflected that they perceived a positive relationship between engaging in earlier post-treatment physical activity and long-term health outcomes compared to survivors who delayed engaging in post-treatment physical activity. It was observed that as survivors advanced their health care literacy regarding breast cancer and lymphedema, a positive shift seemed to occur in self-empowerment and worldview. Making necessary adjustments in perceptions regarding the recovery period also suggested a changing worldview and further emphasized the importance of having adequate social support. Awareness among oncologists and primary care providers of the positive survivorship outcomes associated with earlier referral and use of appropriate resources will further empower survivors to better manage their years of survivorship and any emerging treatment sequelae.

### 4.1. Theoretical Implications

Grounded theory analysis revealed that the relationships between the emerging themes central to long-term breast cancer survivorship were highly inter-connected and may be categorized as psychosocial and cognitive variables. These findings may be best explained by an existing theory of psychoneuroimmunology. Psychoneuroimmunology theory suggests that as the brain perceives and holds thoughts regarding a stressor (e.g., receiving a cancer diagnosis), personal co-factors and psychosocial variables (*i.e.*, religious and spiritual beliefs, social support) mediate the communication via neuro-endocrine pathways between the brain and the immune system [50,51]. Life experiences (e.g., cancer survivorship) influence our thoughts, which changes our beliefs (e.g., spiritual/religious, worldviews), and consequently changes how our brain functions [52]. Moreover, personal co-factors (e.g., age, health care literacy, and expectations of recovery) influence how individuals perceive and filter stressors, cope psychologically, and respond physiologically [52]. Learning from life experiences produces a change in gene expression that alters the patterns of brain nerve cell connectivity; and therefore, psychosocial variables (e.g., social support, worldview, and self-empowerment) can significantly influence the mind’s thought processes [53]. The subsequent adaptive immune system response then manifests as psychological or physiological symptom (disease) or health maintenance (wellness) [54].

### 4.2. Limitations

Participants’ responses were grouped according to participant across time, which may affect the resulting themes, as it was expected that different themes would emerge at different stages of survivorship. For example, those participants who were enrolled at post-op or unable to continue in the study for the full 30 months had fewer opportunities to provide data. Additionally, survivors who participated longer may have had more time to reflect about their survivorship experiences and offer multiple perspectives regarding different themes as their survivorship period progressed. It is noted that, that although there were some differences in the length of time participants were in the study, this was a qualitative study with an emphasis on the substance of views offered across time, rather than the number of times those same views were stated.

### 4.3. Strengths

This was a longitudinal study that spanned 30 months of data collection from the same participants (*N =* 379). Participants were similar with respect to being newly diagnosed with breast cancer, as well as the timing when they entered the study (*i.e.*, diagnosis or within one month post-operative). It is further noted that the parent study spanned 84 months. Overall attrition for the first 30 months was low.

## 5. Conclusions and Implications

Breast cancer survivors continue to have an increased risk for long-term psychological distress due to a lifetime risk of post-treatment related sequela (e.g., lymphedema and recurrence of breast cancer). A psychoneuroimmunological model of health suggests that long-term breast cancer survivorship care planning should consider interventions to reduce stress and psychological distress. Survivors suggested that the two most common factors essential to long-term survivorship were having a strong social support network and cultivation of a positive worldview. Our data were consistent with a growing body of literature that suggests perceptions of social support may be associated with health outcomes in long-term cancer survivorship.

Themes from the data suggested that breast cancer survivors experienced a positive shift in worldview and self-empowerment during the cancer survivorship continuum. Findings suggested that long-term stress may be reduced by interventions that promote positive worldviews, self-empowerment, and spiritual and religious practices. Interventions may include the encouragement of CAM practices (e.g., positive affirmations, meditations, and prayers).

Survivors indicated a desire to better understand their diagnoses and treatment options. Moreover, survivors demonstrated a preference for seeking additional information resources to complement information provided by their health care providers and cancer centers. This suggests that breast cancer and lymphedema education strategies need to consider the non-traditional resources for patient learning (e.g., mentoring programs), as well as the timing and access to educational materials (e.g., interactive web-based programs). In general, data suggested that breast cancer survivors who were self-empowered and able to advocate for themselves throughout the survivorship continuum had more positive perceptions of their health outcomes and post-treatment experiences.
